# Global epistasis in plasmid-mediated antimicrobial resistance

**DOI:** 10.1038/s44320-024-00012-1

**Published:** 2024-02-26

**Authors:** Javier DelaFuente, Juan Diaz-Colunga, Alvaro Sanchez, Alvaro San Millan

**Affiliations:** 1 Centro Nacional de Biotecnología (CNB-CSIC), Madrid, Spain; 2 Institute of Functional Biology & Genomics, IBFG - CSIC, Universidad de Salamanca, Salamanca, Spain; 3 Centro de Investigación Biológica en Red de Epidemiología y Salud Pública (CIBERESP), Instituto de Salud Carlos III, Madrid, Spain

**Keywords:** Global Epistasis, Antimicrobial Resistance, Plasmids, Microbiology, Evolution, Microbiology, Virology & Host Pathogen Interaction

## Abstract

Antimicrobial resistance (AMR) in bacteria is a major public health threat and conjugative plasmids play a key role in the dissemination of AMR genes among bacterial pathogens. Interestingly, the association between AMR plasmids and pathogens is not random and certain associations spread successfully at a global scale. The burst of genome sequencing has increased the resolution of epidemiological programs, broadening our understanding of plasmid distribution in bacterial populations. Despite the immense value of these studies, our ability to predict future plasmid-bacteria associations remains limited. Numerous empirical studies have recently reported systematic patterns in genetic interactions that enable predictability, in a phenomenon known as global epistasis. In this perspective, we argue that global epistasis patterns hold the potential to predict interactions between plasmids and bacterial genomes, thereby facilitating the prediction of future successful associations. To assess the validity of this idea, we use previously published data to identify global epistasis patterns in clinically relevant plasmid-bacteria associations. Furthermore, using simple mechanistic models of antibiotic resistance, we illustrate how global epistasis patterns may allow us to generate new hypotheses on the mechanisms associated with successful plasmid-bacteria associations. Collectively, we aim at illustrating the relevance of exploring global epistasis in the context of plasmid biology.

## Abstract (web summary)

Antimicrobial resistance (AMR) in bacteria is a major public health threat and plasmids play a key role in AMR gene dissemination. This Review discusses the relevance of global epistasis patterns for predicting AMR evolution and generating hypotheses about the mechanisms.

## Graphical abstract

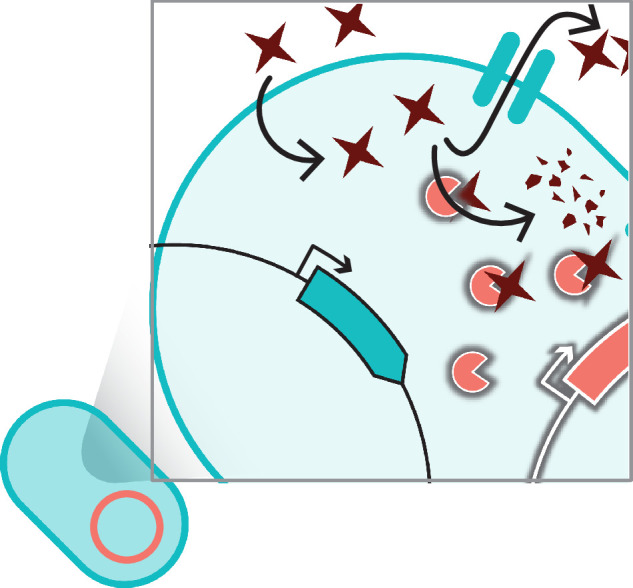

## Epistatic interactions between AMR plasmids and bacteria

Antimicrobial resistance (AMR) is a major public health problem. Recent studies indicate that within a few decades AMR infections could become the leading cause of death worldwide and are set to produce annual losses of $2-6.1 trillion (Jonas Olga et al, 2017; Murray et al, 2022; O’Neill, 2016). AMR is particularly concerning in hospitals, where antibiotic exposure selects for multi-drug resistant (MDR) bacteria. MDR clones frequently colonize hospitalized patients, increasing hospitalization periods and mortality rates (Vincent, 2009). In this context, plasmids play a key role in the evolution and spread of AMR mechanisms. Plasmids are extra-chromosomal genetic elements that can transfer horizontally between bacteria, mainly by conjugation (Rodríguez-Beltrán et al, 2021). In addition, plasmids can transfer vertically coupled to the cell division of the bacterial host. Crucially, conjugative plasmids are the main vehicle for the spread of AMR genes in clinically relevant bacterial populations such as enterobacteria (order *Enterobacterales*) (Partridge et al, 2018).

AMR plasmids are widespread in clinical settings, but their distribution across bacterial hosts is not random (David et al, 2020). Certain associations between clinically important bacterial clones and AMR plasmids are particularly successful and spread at a global scale. Examples of these associations include *Klebsiella pneumoniae* sequence type 11 (ST11) carrying the carbapenem resistance pOXA-48-like plasmids (David et al, 2019; San Millan, 2018) and *Escherichia coli* ST131 carrying IncF plasmids encoding the extended spectrum ß-lactamases CTX-M (Palkovicova et al, 2022; Pitout and Finn, 2020). Colonization by one of these high-risk clones represents a serious threat for hospitalized patients (Tischendorf et al, 2016). In order to predict and control AMR in clinical settings it is imperative to understand the evolutionary and molecular basis underlying the success of plasmid-bacteria associations.

While AMR plasmids allow bacteria to survive in the presence of antibiotics, they can also impose a fitness cost for the host bacteria in the absence of antibiotics (San Millan and MacLean, 2017). Importantly, recent studies have revealed that plasmid-associated phenotypes are variable across bacteria (Alonso-del Valle et al, 2021; Bethke et al, 2020; Cairns et al, 2018; Carrilero et al, 2023; Dimitriu et al, 2019; Fernández-Calvet et al, 2023; Hall et al, 2016; Li et al, 2020). For example, the widespread AMR plasmid pOXA-48 causes different fitness effects and confers different levels of AMR in different wild-type strains of clinical enterobacteria (Alonso-del Valle et al, 2021, 2023; Fernández-Calvet et al, 2023). These epistatic interactions between the plasmid and the genetic background of the host bacterium are a key determinant of the success of plasmid-bacteria associations (Benz and Hall, 2023; Kosterlitz et al, 2023).

Epistatic interactions between plasmids and the genome of their bacterial hosts add ruggedness to the adaptive landscapes of plasmid-mediated AMR and hinder our ability to predict AMR evolution (Wong, 2017). To successfully predict AMR evolution given this complexity, one would have to identify all *loci* in the host’s genome that may epistatically interact with the genetic content of the plasmid and to quantify every possible pairwise and higher order interaction, an imposing and practically impossible task. Encouragingly, recent evidence suggests that the aggregate effect of multiple epistatic interactions can lead to the fitness effect of a mutation following simple, systematic patterns, which enable a certain degree of predictability (Bakerlee et al, 2022; Chou et al, 2011; Johnson et al, 2019; Khan et al, 2011; Kryazhimskiy et al, 2014; MacLean et al, 2010; Otwinowski et al, 2018; Perfeito et al, 2014; Reddy and Desai, 2021; Schoustra et al, 2016; Wei and Zhang, 2019). This emergent phenomenon, known as global epistasis (see Box 1), leads to simple statistical regularities where the fitness effect of a mutation (Δ*F*) can be predicted by the fitness of the background where it is added (*F*_*B*_).

This perspective piece is inspired by the idea that global epistasis provides a useful framework to detect and understand interactions between AMR plasmids and clinical bacteria. Specifically, we propose that global epistasis patterns could provide valuable insights into the evolution of plasmid-mediated AMR. At a practical level, these patterns could help us predict the success of specific plasmid-bacteria associations, bringing us closer to the goal of being able to anticipate AMR evolution in clinical settings. At a more fundamental level, patterns of global epistasis could give clues into the functional relationships between AMR mechanisms and genetic backgrounds, helping us to determine the molecular basis underlying the success of these high-risk associations.

Here, we examine the existence of global epistasis patterns in plasmid-bacteria associations across two different host species. We show that one of the two hosts exhibits such patterns while the other one does not, and appears to be largely dominated by additive effects and idiosyncratic epistasis. For this exercise, we have re-analyzed recently published data (Alonso-del Valle et al, 2021, 2023; Fernández-Calvet et al, 2023). To illustrate how the different patterns of antibiotic resistance could shed light into genetic mechanisms, we explore a simple mechanistic model of plasmid-acquired antibiotic resistance and examine how different types of mechanistic interactions lead to different patterns. Overall, the objective of this piece is to raise awareness of the application of global epistasis to the antibiotic resistance problem and to highlight what we can learn from it.

Box 1 Global epistasis patterns allow us to predict evolutionary pathways Are interactions between mutations predictable? Fitness landscapes map the relationship between genotypes and fitness within specific environmental conditions, helping us to predict evolutionary processes. If interactions between mutations are completely additive, to reconstruct the entire landscape we would only need a small training subset that contains all the mutations. On the other hand, if the associations between mutations and phenotypes were entirely random, no subset of measurements would reveal any pattern. Such interactions between mutations —a phenomenon known as epistasis—create highly complex landscapes and hamper our ability to predict evolution. The term ‘epistasis’ has been widely used over the years by biologists at various scales (Phillips, 2008). Here, we use the term ‘epistasis’ to describe the scenario where the overall fitness resulting from multiple mutations differs from what is expected based on the additive effects of each individual mutation.

Epistatic interactions between mutations are commonly reported in scientific literature and multiples studies have mapped the effect of mutations in different genetic backgrounds (Chou et al, 2011; Johnson et al, 2019; Khan et al, 2011; MacLean et al, 2010; Schoustra et al, 2016; Schubert et al, 2019; Vogwill et al, 2016). Importantly, numerous works have observed that epistatic interactions can exhibit systematic patterns that facilitate predictability (Diaz-Colunga et al, 2023b; Husain and Murugan, 2020; Johnson et al, 2023; Khan et al, 2011; Kryazhimskiy et al, 2014; Lyons et al, 2020; Reddy and Desai, 2021; Schoustra et al, 2016; Wei and Zhang, 2019; Wünsche et al, 2017). Epistatic effects on the phenotype could contain a global component, where the phenotypic impact of a mutation (Δ*F*), can be predicted by the phenotype of the different backgrounds in which it is introduced (*F*_*B*_). This phenomenon is known as global epistasis.

Different patterns of global epistasis have been documented. Beneficial mutations can produce either a greater (*accelerating returns*) or weaker (*diminishing returns*) effect as the fitness of the background increases (Chou et al, 2011; Khan et al, 2011; MacLean et al, 2010; Wang et al, 2016). Similarly, detrimental mutations can have weaker (*diminishing costs*) or stronger (*accelerating costs*) fitness effects as a function of the fitness of the genetic background (Johnson et al, 2019).

**Figure:**
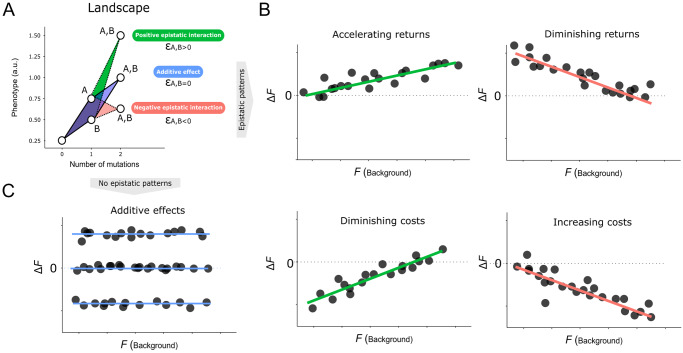
**Global epistasis.** (**A**) We consider a scenario in which the fitness (or phenotype, in *y*-axis) of an organism is defined by the contribution of different mutations in the genome (mutation A, B or both). If there is no interaction between mutations (additive effect, blue in figure), the sum of the effect of both mutations can be predicted from the individual effect of each mutation (ε_A,B_ = 0). On the contrary, if there are interactions between mutations (ε_A,B_ ≠ 0), the effect on the phenotype of the double mutant is less predictable (green and red in figure). (**B**) Possible global epistatic patterns that could arise from mutational interactions at a global scale. Note that now *x*-axis represents the fitness of the different backgrounds (*F*_*B*_) and the *y*-axis represents the fitness effect of a mutation in each different genetic background (Δ*F*). Importantly, each different genetic background could include various mutations (mutations A, B, C, …). The lines represent a simple linear model that correlates the fitness effect of a mutation and the fitness of each background. Green and red lines indicate positive and negative slopes, respectively. (**C**) A scenario in which the effect of the mutation (Δ*F*) is the same in all genetic backgrounds (*F*_*B*_). In this case, each blue line represents the linear model for positive, neutral or negative effects of Δ*F*. Note that there is not a clear pattern of interaction between mutations (slope = 0).

## Screening for epistatic patterns in plasmid-associated phenotypes

The last decade has witnessed a surge of interest in the evolutionary dynamics of plasmid-bacteria associations, producing a great many studies on this topic (Brockhurst and Harrison, 2022; Rodríguez-Beltrán et al, 2021). We have specifically focused on the study of an epidemic group of carbapenemase-encoding plasmids, pOXA-48-like plasmids (from here on pOXA-48). pOXA-48 is a broad-host-range conjugative plasmid from the plasmid taxonomic unit L/M (Pitout et al, 2019; Redondo-Salvo et al, 2020). pOXA-48 encodes the OXA-48 carbapenemase and is disseminated across enterobacteria worldwide (Pitout et al, 2019). Carbapenemases are enzymes able to degrade carbapenem antibiotics, which are last resort antibiotics used in clinical settings to treat MDR infections. Therefore, carbapenemase-producing enterobacteria are a particularly concerning group of hospital-based (nosocomial) bacteria, and are part of the WHO “priority pathogens” list (WHO. 2017). Over the last years, we have studied hundreds of associations between pOXA-48 and clinical enterobacteria recovered from the gut microbiota of hospitalized patients (Alonso-del Valle et al, 2021, 2023; DelaFuente et al, 2022; Fernández-Calvet et al, 2023; León-Sampedro et al, 2021). We have characterized the epidemiology and evolution of pOXA-48-mediated resistance in a hospital (DelaFuente et al, 2022; León-Sampedro et al, 2021), as well as different pOXA-48-associated phenotypes, such as conjugation ability, fitness effects, and AMR levels, across a wide range of bacterial hosts (Alonso-del Valle et al, 2021, 2023; Fernández-Calvet et al, 2023).

To examine whether the pOXA-48-mediated resistance is shaped by global epistasis, we used two collections of clinical enterobacteria that were isolated from the same cohort of patients (Alonso-del Valle et al, 2021; Fernández-Calvet et al, 2023). The first collection included strains that did not carry pOXA-48 when they were recovered from the gut microbiota of patients (Alonso-del Valle et al, 2021). The second collection included pOXA-48-carrying strains (Fernández-Calvet et al, 2023). For both collections we constructed pOXA-48-carrying and pOXA-48-free isogenic versions of each strain by introducing pOXA-48 by conjugation (Alonso-del Valle et al, 2021) or by selectively removing pOXA-48 with a CRISPR-Cas9-based tool (DelaFuente et al, 2022; Fernández-Calvet et al, 2023). Bacteria in these collections belong to the two most prevalent species associated with pOXA-48 in hospitals, *Klebsiella* spp. and *E. coli*. In the *Klebsiella* spp. group we included isolates from three species: *K. pneumoniae*, *K. quasipneumoniae* and *K. variicola*, which are also associated with pOXA-48 in our hospital (DelaFuente et al, 2022; León-Sampedro et al, 2021). Altogether, the data generated in those studies provide a unique opportunity to perform a high-throughput analysis of pOXA-48-bacteria interactions.

### Plasmid-associated fitness effects

**Figure 1:**
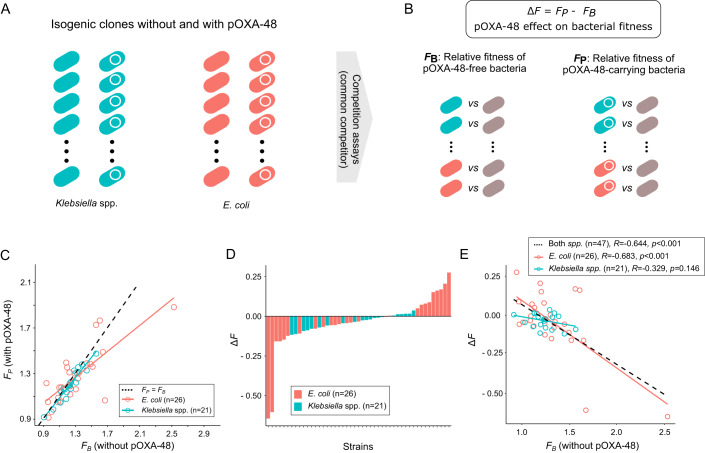
Global epistasis trends for plasmid-associated fitness effects in the absence of antibiotics. (**A**) In our previous work (Alonso-del Valle et al, 2021; Fernández-Calvet et al, 2023), we generated a collection of isogenic pairs of pOXA-48-carrying/pOXA-48-free bacteria isolated from the gut of hospitalized patients. (**B**) In the same studies, we performed competition assays against a common *E. coli* strain to determine the relative fitness of each pOXA-48-carrying (*F*_*P*_) and pOXA-48-free (*F*_*B*_) clones in the absence of antibiotics. Using these data, we calculated pOXA-48 effects in bacterial fitness (Δ*F*). (**C**) Comparison of the relative fitness of the pOXA-48-carrying and pOXA-48-free strains (*F*_*P*_ and *F*_*B*_, respectively). The red and blue lines indicate the correlation for *E. coli* and *Klebsiella* spp., respectively. The black dashed line corresponds to the line when *F*_*P*_ equals *F*_*B*_ (y = x). (**D**) Δ*F* distribution for all strains. Species are indicated by colors (blue for *Klebsiella* spp. and red for *E. coli*). Note that *Klebsiella* spp. strains exhibit lower variability of pOXA-48 fitness effects than *E. coli* strains. (**E**) We represent the fitness effects produced by pOXA-48 (Δ*F*) as a function of the relative fitness of the pOXA-48-free background (*F*_*B*_). Interestingly, we observed that Δ*F* and *F*_*B*_ correlated negatively in *E. coli* but not in *Klebsiella* spp. (Pearson correlation, *R* = −0.683, *p* < 0.001, sample size = 26 and *R* = −0.329, *p* = 0.146, sample size = 21, respectively).

Relative fitness is the first phenotype we analysed. In previous work and for many of the strains in our collections, we independently competed pOXA-48-free and pOXA-48-carrying clones against a common competitor (a GFP-labeled laboratory strain of *E. coli*) in the absence of antibiotics (Alonso-del Valle et al, 2021; Fernández-Calvet et al, 2023). This way we could determine the relative fitness of each pOXA-48-carrying and pOXA-free strain against the common competitor (*F*_*P*_ *and F*_*B*_, respectively, see Fig. 1A–C), as well as calculate the change in relative fitness associated with carrying pOXA-48 in each background (Δ*F*) (Fig. 1D,E).

Our results indicated that pOXA-48 produced a wide range of fitness effects across bacterial hosts (Fig. 1D), and that plasmid fitness costs become larger as the fitness of the background increases (Pearson correlation coefficient *R* = −0.644*, p* < 0.001, sample size = 47, black dashed line in Fig. 1E). When we divided the data by genus, we observed that the *increasing costs epistasis* was driven by *E. coli* strains, while no significant trend was observed in *Klebsiella* spp. strains (Pearson correlation, *R* = −0.683*, p* < 0.001, sample size = 26 and *R* = −0.329, *p* = 0.146, sample size = 21, respectively). It is important to note that although the fitness values of the pOXA-48-free *Klebsiella* spp. strains show remarkable variability (ranging between 0.911 and 1.561), the fitness effect of pOXA-48 is quite consistent across these strains (Δ*F* = −0.032 ± 0.045, sample size = 21) compared to in *E. coli* (Δ*F* = −0.042 ± 0.207, sample size = 26).

### Plasmid-mediated AMR

**Figure 2:**
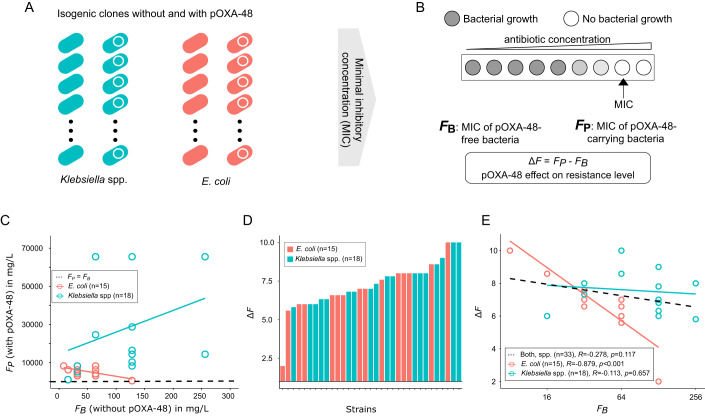
Global epistasis trends for plasmid-mediated AMR. (**A**) In ref (Alonso-del Valle et al, 2023), we generated a collection of isogenic pairs of pOXA-48-carrying/pOXA-48-free bacteria isolated from the gut of hospitalized patients. (**B**) To measure antibiotic resistance levels, we determined the minimal inhibitory concentration (MIC) of amoxicillin-clavulanate (AMC) for each clone. MIC is defined as the lowest antibiotic concentration that inhibits bacterial growth. We determined the MIC values for pOXA-48-free strains (*F*_*B*_) and for the same strains carrying pOXA-48 (*F*_*P*_), and we calculated the change in AMC resistance level associated with pOXA-48 acquisition for each strain (Δ*F*). (**C**) Comparison of the resistance level to amoxicillin-clavulanate (AMC) in mg/L of different isogenic pairs of pOXA-48-carrying and pOXA-48-free enterobacteria (*F*_*P*_ and *F*_*B*_, respectively). The red and blue lines indicate the correlation for *E. coli* and *Klebsiella* spp., respectively. The black dashed line corresponds to the line when *F*_*P*_ equals *F*_*B*_ (y = x). (**D**) Distribution of Δ*F* for all strains measured as fold change in MIC levels (log_2_MIC *F*_*P*_ - log_2_MIC *F*_*B*_). Species are indicated by colors (blue for *Klebsiella* spp. and red for *E. coli*). (**E**) We represent the effect of pOXA-48 in AMC resistance (Δ*F*) as a function of the MIC in the pOXA-48-free bacterial background (*F*_*B*_). *F*_*B*_ (*X*-axis) is represented in log_2_ scale and Δ*F* (*Y*-axis) is calculated as (log_2_MIC *F*_*P*_ - log_2_MIC *F*_*B*_). Again, we observed a negative correlation between Δ*F* and *F*_*B*_ in *E. coli* but not in *Klebsiella* spp. (Pearson correlation; *R* = −0.879, *p* < 0.001, sample size = 15 and *R* = −0.113, *p* = 0.657, sample size = 18, respectively).

The second phenotype analyzed for global epistatic trends was AMR. We previously characterized the AMR levels conferred by pOXA-48 in a collection of 50 wild-type *Klebsiella* spp. and *E. coli* strains isolated from patients, which were pOXA-48-free at the time of isolation (Alonso-del Valle et al, 2023). Most of the strains in this study are the same previously used to determine pOXA-48 fitness effects in the absence of antibiotics (Alonso-del Valle et al, 2021). We first determined the resistance level of each pOXA-48-carrying/pOXA-48-free bacterial pair to amoxicillin-clavulanate (AMC, *F*_*P*_ and *F*_*B*_, respectively, see Fig. 2A–C), a commonly used β-lactam therapy in hospitals. To measure the AMR level, we used the minimal inhibitory concentration (MIC). MIC is defined as the lowest antibiotic concentration that inhibits bacterial growth and is extensively used in clinical microbiology to determine the AMR phenotypes of clinical bacteria (EUCAST. 1998). Again, similarly to the fitness effect distribution (Fig. 1D), the level of AMC resistance conferred by pOXA-48 (Δ*F*) was variable across clones (Fig. 2D). In Fig. 2E, we represent the fold-change in AMC resistance level associated with acquiring pOXA-48 (Δ*F*) compared to the AMC resistance level of the plasmid-free strain (*F*_*B*_), and we observed no clear trend. However, when the data was divided by genus, we observed a clear pattern of *diminishing returns epistasis* for *E. coli* strains, but not for *Klebsiella* spp. strains.

## Global epistasis may point to hypotheses regarding mechanisms of plasmid-bacteria interactions

Our results indicate that the fitness effect of a plasmid can strongly depend on its genetic background: in the absence of antibiotic, pOXA-48 can be beneficial in some *E. coli* strains but detrimental in others, and the level of resistance it confers is also rather variable. This indicates the existence of strong genetic interactions between plasmid-encoded genes and those in the host genome. Given the massive size of the genome and the astronomically high number of such potential interactions, predicting the fitness effects of the plasmid in any given strain seems incredibly difficult. However, the emergence of global epistasis patterns partly alleviates this problem, indicating that it is important to pay attention to these patterns. Besides these potential practical implications, global epistasis patterns may also help us identify the mechanisms of interaction between plasmid and genome. Recent theoretical work has established that the slope of global epistasis patterns is mathematically connected to the “microscopic” pairwise epistatic interactions between the plasmid and every locus in the genome, through this Eq. (1):

1 $$b \approx \mathop {\sum}\limits_i {\tilde \varepsilon _{ip}} $$

where *b* is the slope of the global epistasis regression, the sum goes over all *loci*, and $\tilde \varepsilon _{ip}$ captures the interaction between locus *i* and the plasmid (*p*) (Diaz-Colunga et al, 2023b; Reddy and Desai, 2021). Although Eq. (1) neglects high-order epistatic interactions, it provides a good approximation in many empirical systems (Diaz-Colunga et al, 2023a, b; Reddy and Desai, 2021).

What does this mean, in practice? Under certain simplifying assumptions, negative regression slopes suggest that the aggregate effect of all negative epistatic interactions is stronger than that of positive epistatic interactions. On the other hand, positive regression slopes may suggest that the aggregate effect of positive epistasis dominates over negative epistasis. Flat, near-zero slopes may simply indicate additive fitness effects and low epistasis, but may also indicate that positive and negative epistatic interactions balance each other out. Of course, the math has subtleties that must be carefully weighed and there may be alternative explanations for those patterns. Our goal here is not to cover these details in depth, as this has been done in recent reviews and theoretical work (Bakerlee et al, 2022; Diaz-Colunga et al, 2023b; Johnson et al, 2023; Reddy and Desai, 2021). Nevertheless, the existence of negative regression slopes between the fitness effect of a plasmid and the fitness of the plasmid-free strain may point out to specific molecular and genetic mechanisms of interactions, leading to hypotheses that are worth testing independently.

To illustrate this point, we use a simple mathematical model of antibiotic resistance (see Box 2) that incorporates passive antibiotic diffusion in and out of the cell as well as various molecular mechanisms of antibiotic resistance (enzymatic inactivation, pumping, etc.). This is arguably the simplest quantitative model of intracellular antibiotic dynamics, and it has been studied in different forms in previous work (Artemova et al, 2015; Yurtsev et al, 2013). In the context of this model, we assume that different plasmid-free strains may harbor a variety of endogenous resistance mechanisms that confer some protection against the antibiotic (see Box 2). We then consider two alternative scenarios: one where the plasmid encoded resistance mechanisms overlap with those endogenous to the plasmid-free strain (i.e. they are partly redundant), and one where the resistance mechanisms do not overlap. In the first such case, we observe negative (i.e. sub-additive) pairwise epistasis between the plasmid and the mechanistically overlapping resistance genes in the host. When we examine the fitness effects of the plasmid on different strains, we see that the more resistant the background strain, the smaller the effect of the plasmid. By contrast, when the plasmid is introduced in strains with little overlap between the endogenous and the plasmid encoded resistance mechanisms, the fitness effect of the plasmid in different strains are not necessarily correlated with the fitness of the host. However, if the plasmid-encoded mechanism is complementary with the endogenous one, then positive epistasis may arise between them.

We would like to emphasize that we do not claim that whenever a negative correlation is observed, this by defaults means an overlap in resistance mechanisms, or that the absence of such correlation necessarily indicates absence of mechanistic overlap. It is important to keep in mind that alternative explanations exist. For instance, a negative correlation can arise as a result of antagonistic epistasis with the host’s endogenous resistance mechanisms, and lack of correlation may arise when the plasmid contains genes that interact synergistically with endogenous mechanisms but other (or the same) genes that interact antagonistically with the endogenous mechanisms. The statistical significance of the correlations must be established carefully, as they may just be artifactual. Our point is not that the mechanism can directly be revealed by global epistasis patterns, but rather that these patterns may be used to generate hypotheses that could be then tested by independent means.

Box 2 Simple mathematical models can guide the search for mechanisms governing global epistasis in plasmid-mediated AMR Which mechanisms underlie the emergence of global epistasis patterns in plasmid-mediated AMR? In this Box we present a simple dynamical model from which global epistasis-like patterns can readily emerge when fitness is determined by the intracellular concentration of an antibiotic. We call [*A*_out_] and [*A*_in_] the extracellular and intracellular concentrations of antibiotic, respectively. We consider the extracellular concentration to be constant (as in a chemostat). In the absence of any active mechanisms for antibiotic degradation, inactivation, and/or externalization, the dynamics of [*A*_in_] would be given by *d*[*A*_in_]/*dt* = *v*_0_([*A*_out_] − [*A*_in_]); such that the intracellular concentration of antibiotic approaches the extracellular at a rate controlled by the parameter *v*_0_.

We now consider an active mechanism through which cells may reduce the intracellular concentration of antibiotic, for instance degradation mediated by an enzyme *E*. Assuming degradation follows Michaelis-Menten dynamics, the dynamics of [*A*_in_] would now be given by:

2 $$\frac{{d\left[ {A_{{{{{{{{\mathrm{in}}}}}}}}}} \right]}}{{dt}} = \begin{array}{c}{{v_0\left( {\left[ {A_{{{{{{{{\mathrm{out}}}}}}}}}} \right] - \left[ {A_{{{{{{{{\mathrm{in}}}}}}}}}} \right]} \right)}}\\ {{{{{{{{\mathrm{passive}}}}}}}}\;{{{{{{{\mathrm{internalization}}}}}}}}}\end{array} - \begin{array}{c}{{v_1\left[ E \right]\frac{{\left[ {A_{{{{{{{{\mathrm{in}}}}}}}}}} \right]}}{{K_1 + \left[ {A_{{{{{{{{\mathrm{in}}}}}}}}}} \right]}}}}\\ {{{{{{{{\mathrm{enzymatic}}}}}}}}\;{{{{{{{\mathrm{degradation}}}}}}}}}\end{array}$$

where [*E*] is the concentration of enzyme, *v*_1_ [*E*] is the maximum rate of the reaction, and *K*_1_ determines the value of [*A*_in_] at which the reaction reaches half-maximum rate.

As the system goes to steady state (*d*[*A*_in_]/*dt* = 0), the intracellular concentration of antibiotic approaches a value determined by [*A*_out_], [*E*], and the parameters *v*_0_, *v*_1_ and *K*_1_. In general, we may expect fitness *F* to be a monotonically decreasing function of this intracellular concentration of antibiotic at equilibrium, such as:

3 $$F = \frac{{F_{\max }}}{{K_{{{{{{{\mathrm{F}}}}}}}} + \left[ {A_{{{{{{{{\mathrm{in}}}}}}}}}} \right]}}$$

Note that Eq. 3 above is formulated for illustrative purposes only, and in general the relationship between *F* and [*A*_in_] may take different functional forms.

Within this model, the fitness of a particular strain will be given by its level of constitutive expression of the enzyme *E* (assuming no variation in *v*_0_, *v*_1_, *K*_1_ or *K*_F_ across strains). If a strain acquires a plasmid that encodes the same enzyme *E*, it may increase its expression and further reduce the intracellular concentration of antibiotic, resulting in a positive fitness effect of the plasmid. Note, however, that fitness does not increase linearly with [*E*], and so this positive fitness effect can be expected to be smaller in strains that can naturally express high levels of the enzyme— i.e., those strains with higher resistance levels in the absence of the plasmid. Thus, redundant expression of the enzyme can result in a negative correlation between the fitness effect of the plasmid and the fitness of the plasmid-free strain.

To illustrate this idea, we generated an in silico library of 50 strains, each with a different level of expression of *E*, sampled from a normal distribution (see “Model parameters” below). We then considered that acquiring the plasmid resulted in an increase in *E* expression (varying slightly from strain to strain). We used Eqs. 2 and 3 to quantify the fitness of the plasmid-free and plasmid-carrying strains. In the figure in this Box, we show that, in line with our reasoning, diminishing returns epistasis emerges in the form of a negatively sloped correlation between the fitness of the genetic background and the fitness effect of the plasmid.

A similar model structure can lead to the emergence of different patterns of global epistasis. For instance, we can now consider two potential mechanisms by which a cell may reduce its intracellular concentration of antibiotic: enzymatic degradation or antibiotic externalization via an active pump. The dynamics of [*A*_in_] would now be given by:

4 $$\frac{{d\left[ {A_{{{{{{{{{{\mathrm{in}}}}}}}}}}}} \right]}}{{dt}} = \begin{array}{c} {{v_0\left( {\left[ {A_{{{{{{{{{{\mathrm{out}}}}}}}}}}}} \right] - \left[ {A_{{{{{{{{{{\mathrm{in}}}}}}}}}}}} \right]} \right)}}\\ {{{{{{{{{{\mathrm{passive}}}}}}}}}}\;{{{{{{{{{\mathrm{internalization}}}}}}}}}}}\end{array} - \begin{array}{c} {{v_1\left[ E \right]\frac{{\left[ {A_{{{{{{{{{{\mathrm{in}}}}}}}}}}}} \right]}}{{K_1 + \left[ {A_{{{{{{{{{{\mathrm{in}}}}}}}}}}}} \right]}}}}\\ {{{{{{{{{{\mathrm{enzymatic}}}}}}}}}}\;{{{{{{{{{\mathrm{degradation}}}}}}}}}}}\end{array} - \begin{array}{c} {{v_2\left[ P \right]\frac{{\left[ {A_{{{{{{{{{{\mathrm{in}}}}}}}}}}}} \right]}}{{K_2 + \left[ {A_{{{{{{{{{{\mathrm{in}}}}}}}}}}}} \right]}}}}\\ {{{{{{{{{{\mathrm{pumping}}}}}}}}}}}\end{array}$$

where [*P*] represents the concentration of pumps in the cell wall. We now consider another set of plasmid-free strains which cannot express the enzyme ([*E*] = 0) but can alleviate the intracellular concentration of antibiotic is via the expression of the pump ([*P*] > 0). Under high external antibiotic pressure (high [*A*_out_]), pumps may be saturated (when [*A*_in_] ≫ *K*_2_). In this scenario, acquiring a plasmid which encodes the enzyme *E* may open a new, complementary way for cells to reduce the intracellular levels of antibiotic and increase fitness. This, in turn, may de-saturate the pumps, resulting in a synergistic effect of both mechanisms.

This synergy can make it so the fitness effect of the plasmid is now larger in higher-fitness genetic backgrounds - i.e., in those strains with higher constitutive levels of pump expression, where de-saturating this mechanism results in more substantial fitness gains. To illustrate this idea, we generated a new library of in silico plasmid-free strains (now considering [*E*] = 0 and [*P*] > 0 for all of them), under a stronger external antibiotic pressure ([*A*_out_] ten times larger than in the previous model). Like before, we consider that the enzyme *E* is encoded in the plasmid, so the plasmid-carrying strains can now express it ([*E*] > 0). Using Eqs. 2 and 3 to quantify the fitness of the plasmid-free backgrounds and their plasmid-carrying counterparts, we now see a positive correlation between the background fitness and the fitness effect of the plasmid (*accelerating returns* epistasis).

**Model parameters**

**Model 1: diminishing returns epistasis**. In arbitrary units of time^-1^: *v*_0_ = 1, *v*_1_ = 1. In arbitrary units of fitness: *F*_max_ = 1. In arbitrary units of concentration: [*A*_out_] = 100, *K*_1_ = 50, *K*_F_ = 50, [*E*] randomly sampled from a normal distribution with mean 250 and standard deviation 50 for the genetic backgrounds; the plasmid increases [*E*] by an amount sampled from a normal distribution with mean 100 and a standard deviation of 10.

**Model 2: accelerating returns epistasis**. We now consider a higher external antibiotic pressure: [*A*_out_] = 1000 (in arbitrary units of concentration). We take *v*_2_ = 1 (arbitrary units of time^-1^); *K*_2_ = 50 and [*E*] = 0 (arbitrary units of concentration) for all genetic backgrounds; [*P*] is randomly sampled from a normal distribution with mean 250 and standard deviation 50 for the genetic backgrounds. As in the previous model, the plasmid increases [*E*] by an amount sampled from a normal distribution with mean 100 and a standard deviation of 10. All other parameter values are the same as in the previous model.

**Figure:**
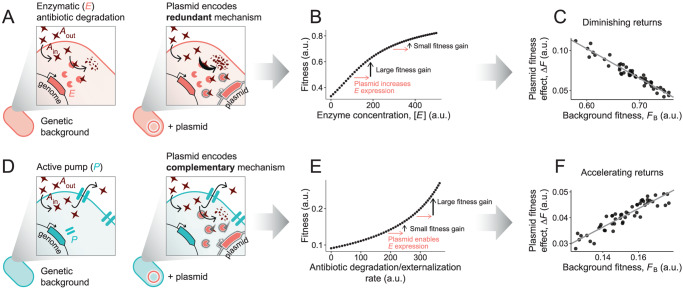
**Global epistasis emerges in a simple model of intracellular antibiotic dynamics.** We consider an antibiotic *A* which can passively enter the cell, and which can be degraded via the action of an enzyme *E* or externalized via the action of a pump *P*. We consider fitness to be a monotonically decreasing function of the intracellular concentration of antibiotic, [*A*_in_]. (**A**) Here, the plasmid encodes the enzyme *E*, which is also encoded in the genome of the background strain. (**B**) Redundant expression of the enzyme results in sub-linear fitness gains: the plasmid has a smaller beneficial fitness effect in background strains which are naturally able to express higher levels of the enzyme (and thus have higher fitness without the plasmid). (**C**) This leads to the fitness effect of the plasmid exhibiting a pattern of *diminishing returns* global epistasis. (**D**) In this alternative scenario, the background strain expresses the pump *P* (we consider a case where the pump operates near saturation). Acquiring the plasmid allows the strain to express the enzyme *E*, in turn alleviating the pump load. (**E**) Both mechanisms act synergistically, so the plasmid is more beneficial in backgrounds with a higher the pump activity. (**F**) This results in a global epistasis pattern of *accelerating returns*.

## Conclusions and outlook

AMR is a critical and difficulty to solve public health problem. The most straightforward solution to the rise of AMR is introducing new antibiotics in clinical practice. However, the known shortage of new antibiotics, combined with the steady increase in antimicrobial resistant infections, complicates the problem further. Therefore, there is an urgent need to implement new approaches to deal with AMR. Improving our ability to predict AMR evolution is key. For example, being able to predict which associations between AMR plasmids and high-risk bacterial clones will emerge in clinical settings would help us to anticipate AMR evolution and, hopefully, to develop more rational intervention strategies to counteract it. We propose that studying global epistasis patterns associated with plasmid-mediated AMR can help us to achieve this goal.

To illustrate this idea, we have re-analyzed data generated in previous studies with clinical enterobacteria carrying plasmid pOXA-48 (Alonso-del Valle et al, 2021, 2023; Fernández-Calvet et al, 2023). Theoretically, any phenotype could be utilized to explore potential patterns of global epistasis as long as the phenotype is measurable before and after plasmid acquisition. In this context, we used these previously published datasets, to screen for patterns of global epistasis affecting two key plasmid-associated phenotypes: pOXA-48 fitness effects in the absence of antibiotics and pOXA-48-mediated AMR levels (Figs. 1, 2).

We measured pOXA-48-associated fitness costs in the absence of antibiotics by using competition assays, a gold standard technique in evolutionary microbiology (Chou et al, 2011; DelaFuente et al, 2020; Khan et al, 2011). However, alternative proxies for bacterial fitness, like growth rates, offer simpler experimental setups and could also be considered (Diaz-Colunga et al, 2023a; MacLean et al, 2010; Schoustra et al, 2016). Similarly, we quantified pOXA-48-mediated AMR levels using MICs. AMR determination by MICs is a widely used method in clinical microbiology that provides quantitative in vitro measurements of AMR levels. However, alternative metrics, such as growth dynamics at subinhibitory antibiotic concentrations, could also be used to investigate the underlying epistatic patterns of AMR.

We observed global epistatic patterns emerging for *E. coli* clones, manifested in negative correlations between the fitness effect of the plasmid and the fitness of the background clones for both phenotypes. For *Klebsiella* spp., on the other hand, we observed no significant correlations. What may be the epidemiological relevance of these observations? One crucial aspect in plasmid-mediated AMR evolution is that, even if plasmids may explore a wide range of bacterial hosts, only a handful of plasmid-bacteria associations tend to succeed and spread in clinical settings. For example, despite pOXA-48 presenting a wide host range and being able to replicate in many enterobacteria species, it is usually associated with a very restricted number of *K. pneumoniae* clones (David et al, 2019, 2020). The results observed here may help to explain this bias in pOXA-48 distribution. Specifically, the probability of pOXA-48 generating a high fitness clone able to spread in a clinical setting both in the presence and absence of antibiotics seems higher for *Klebsiella* spp. clones than for *E. coli* clones, which are constrained by negative epistatic interactions.

Importantly, apart from helping to predict AMR evolution, global epistasis can also enable us to generate hypotheses for the molecular basis underlying plasmid-bacteria interactions. As we introduced in Box 2, redundancy between genomic and plasmid-encoded AMR mechanisms may lead to diminishing return epistatic patterns, similar to those observed in *E. coli*. Incidentally, one key difference between the isolates analyzed in this study is that *E. coli*, but not *Klebsiella* spp., carries a chromosomal *ampC* gene coding for the AmpC ß-lactamase, which confers AMC resistance (Peter-Getzlaff et al, 2011). In *E. coli*, *ampC* is expressed constitutively at a low level, and although its expression is not inducible as in other enterobacteria (Honore et al, 1986; Jacoby, 2009; Peter-Getzlaff et al, 2011), mutations can lead to *ampC* overexpression (Caroff et al, 1999; Olsson et al, 1983; Siu et al, 2003). It is therefore tempting to speculate that the redundancy between AmpC and OXA-48 in terms of AMC hydrolysis could determine the negative epistatic trend observed for AMC resistance levels. Although we realize that this is only one out of many potential explanations for this pattern, we argue that this is an illustrative example of how global epistasis patterns may help to generate testable hypotheses regarding the molecular mechanisms underlying plasmid-bacteria interactions. This could prove highly valuable, considering the wealth of clinical data collected over recent decades, including genomic sequences, bacterial antibiotic resistance profiles, and associated metadata. Analyzing global epistasis patterns within these extensive datasets has the potential to uncover new mechanistic insights associated with the emergence of specific MDR clones.

### Supplementary information

Dataset_EV1

## Data Availability

The data re-analyzed in this study is available in Dataset EV1.
